# Therapeutic Approaches Targeting MYC-Driven Prostate Cancer

**DOI:** 10.3390/genes8020071

**Published:** 2017-02-16

**Authors:** Richard J. Rebello, Richard B. Pearson, Ross D. Hannan, Luc Furic

**Affiliations:** 1 Prostate Cancer Translational Research Laboratory, Peter MacCallum Cancer Centre, Melbourne, VIC 3000, Australia; richard.rebello@monash.edu; 2 Cancer Program, Biomedicine Discovery Institute and Department of Anatomy & Developmental Biology, Monash University, Melbourne, VIC 3800, Australia; 3 Oncogenic Signalling and Growth Control Program, Peter MacCallum Cancer Centre, Melbourne, VIC 3000, Australia; 4 Sir Peter MacCallum Department of Oncology, University of Melbourne, Parkville, VIC 3010, Australia; 5 Department of Biochemistry and Molecular Biology, University of Melbourne, Parkville, VIC 3010, Australia; 6 Department of Biochemistry and Molecular Biology, Monash University, Melbourne, VIC 3800, Australia; 7 The ACRF Department of Cancer Biology and Therapeutics, The John Curtin School of Medical Research, The Australian National University, Acton, ACT 2601, Australia; 8 School of Biomedical Sciences, University of Queensland, Brisbane, QLD 4072, Australia

**Keywords:** MYC, therapy, prostate cancer, mouse models

## Abstract

The transcript encoding the proto-oncogene MYC is commonly overexpressed in prostate cancer (PC). MYC protein abundance is also increased in the majority of cases of advanced and metastatic castrate-resistant PC (mCRPC). Accordingly, the MYC-directed transcriptional program directly contributes to PC by upregulating the expression of a number of pro-tumorigenic factors involved in cell growth and proliferation. A key cellular process downstream of MYC activity is the regulation of ribosome biogenesis which sustains tumor growth. MYC activity also cooperates with the dysregulation of the phosphoinositol-3-kinase (PI3K)/AKT/mTOR pathway to promote PC cell survival. Recent advances in the understanding of these interactions through the use of animal models have provided significant insight into the therapeutic efficacy of targeting MYC activity by interfering with its transcriptional program, and indirectly by targeting downstream cellular events linked to MYC transformation potential.

## 1. Prostate Cancer

The prostate gland surrounds the base of the male bladder and is highly prone to tumorigenesis in aged men. Prostate cancer (PC) is now the most commonly diagnosed male cancer and is a leading cause of death in men aged 40–70 [1,2]. The burden of PC in the United States is significant, with an estimated annual morbidity of over 180,000 men while PC-related mortality exceeds 26,000 men annually [1,3]. Therefore, there has been significant focus on therapeutic strategies targeting the most relevant oncogenic disease drivers which remain active in therapy-resistant PC [3].

PC diagnosis is achieved through identification of pronounced histological features known as Gleason patterns which correlate with aggressiveness of the disease and survival [4]. Developing models recapitulating the histological features of human PC has been a priority for the past two decades. The adult human prostate is regionally divided into zones [5]. The outermost peripheral zone comprises over 70% of prostate glandular tissue and as men age this is the most common region for tumourigenesis [5,6]. Within this site, the normal ductal architecture of the prostate consists of a number of different cell types; luminal and basal epithelium surrounded by fibromuscular stroma (fibroblasts, myocytes and endothelial cells) and neuroendocrine cells [7,8]. The luminal epithelium in PC is thought to undergo gradual transformation through a series of stages of progression from benign disease to adenocarcinoma [9,10,11]. Pre-malignant lesions known as prostatic intraepithelial neoplasia (PIN) are often seen before onset of PC or as concurrent pathology and are thought to be precursors to malignant disease [12]. This pathology is characterized by hyper-proliferation of luminal epithelial cells with a corresponding reduction in the number of basal epithelial cells. These hyperplastic cells have characteristically enlarged nuclei, cytoplasmic hyperchromasia and nuclear atypia [13]. The degree of PIN varies from low-grade PIN (LGPIN) to high-grade PIN (HGPIN) where HGPIN lesions display multilayered luminal epithelium with greater elevation of proliferative markers such as Ki-67 and increased mitotic figures, as observed by phosphorylated histone H3 staining (p-HH3) [14,15]. PC foci often contain histologically heterogeneous lesions [16], however more than 95% of all prostate tumors originate from ductal epithelium and are classed as adenocarcinoma [17]. Diagnosis and scoring of adenocarcinoma is done by visual assessment according to Gleason pattern [4,18] but further assessment can be done by immunostaining mounted sections with basal epithelial marker p63 or cytokeratin 5/14, where the loss of basal cells around the luminal periphery are indicative of adenocarcinoma [10]. Luminal epithelial markers which are commonly over-expressed in carcinomas are also used to detect prostate adenocarcinoma such as α-methylacyl-CoA racemase (AMACR) [10] and cytokeratin 8/18 [19,20]. Recurrent disease post-androgen ablation therapy is often characterized by persistent tumor growth in a low testosterone environment with cells expressing varied levels of functional androgen receptor (AR). This pathology is termed castrate-resistant prostate cancer (CRPC) [2]. Therapy-induced subtypes of recurrent PC are also inherently resistant to hormone ablation [21], Neuroendocrine prostate cancer (NEPC) is classically insensitive to most conventional therapies which target the androgen receptors [22]. NEPC was previously an uncommon pathology only making up less than 2% of all cases of PC, however it is becoming a more frequent observation, likely emerging from hormone-sensitive adenocarcinoma as a consequence of hormone ablation [22,23]. Characteristically, NEPCs express high levels of synaptophysin and chromogranin A, and these markers are commonly used to stratify subtypes of PC and direct therapy accordingly [21].

## 2. MYC in Prostate Cancer

*c-MYC* (*MYC*) is a well-established oncogene and along with family members MYCN and MYCL are significant in driving tumorigenesis in prostate cancer. MYC was first described as a key driver of Burkitt’s lymphoma [24,25] but is now known to have wider influence in other hematological and solid tumors [26]. Accordingly, it is well documented that PC tumor foci show marked overexpression of *MYC* mRNA [27,28] and protein [29] and this correlates with increased disease severity [15,30]. MYC overexpression in aggressive disease is associated with common gene alterations, particularly TMPRSS2-ERG gene fusion [31] which is seen in almost 60% of prostate cancers. Chromosomal amplification of region 8q24, which harbors the *MYC* gene, is also frequently mutated in PC [32,33]. Interestingly, MYCL and MYCN appear to be differentially expressed based on clinical stage and can be mutually exclusive to the expression of MYC [34]. MYCL amplification has recently been shown in Gleason 7 patient biopsies, detected in premalignant lesions and early tumor foci alongside MYC amplification [34]. In contrast, MYCN appears exclusively in aggressive CRPC, found to be overexpressed in up to 40% of NEPC [21,35]. Strikingly, it was recently shown that MYCN-driven mouse models recapitulate the hormone-insensitive human neuroendocrine phenotype [36,37].

MYC promotes oncogenic signaling in PC [26] and stimulates cell growth by binding ribosomal DNA (rDNA) directly and activating ribosomal RNA synthesis [38,39,40]. Significantly, this action activates rDNA transcription via chromatin remodeling and by enhancing upregulation of co-factors for RNA Polymerase (RNA Pol) I pre-initiation complex assembly [38,40]. Here, MYC recruits the selectivity promotor factor (SL1) to the rDNA promotor [38,41,42] and upregulates upstream binding factor (UBF) transcription and recruitment directly [43], leading to efficient pre-RNA synthesis. Accordingly, increased rRNA is frequently observed in PC and associated alterations in prostate epithelial nucleolar structure are potentially MYC directed, as they have been also observed in MYC-driven mouse models [44,45]. MYC family proteins are also implicated in the pro-tumorigenic epigenetic activation of genes in PC. Both MYC [46] and MYCN [37] directly upregulate enhancer of zeste homolog 2 (EZH2) which is linked to prostate cancer progression. This is achieved by suppression of tri-methylated lysine 27 of histone H3 (H3K27me3) [47]. Given this recent revelation, MYC/MYCN-dependent PC may be particularly sensitive to treatment with selective EZH2 inhibitors, some of which are in clinical trials to treat other malignancies [48].

Additionally, in PC it has been shown that MYC overexpressing cells have suppressed tumor suppressor gene (TSG) function. The prostate-specific homeobox NKX3.1 transcription factor [49,50] which in tissue homeostasis competes for MYC target genes and suppresses MYC activity is a powerful example [49]. Loss of function of NKX3.1 is frequently observed in PC [51]. Overexpression of MYC has been demonstrated experimentally to reduce NKX3.1 expression in numerous mouse models [52,53,54] including MYC-driven models [50]. The bridging integrator 1 (BIN1) protein, originally identified as a MYC-interacting tumor suppressor [55], is frequently missing in primary prostate adenocarcinoma, where MYC is deregulated [56]. In cooperation with MYC-interacting zinc finger transcription factor 1 (MIZ1), MYC transcriptionally represses BIN1 promoter [57], suggesting a positive feedback loop of MYC deregulation by inhibiting its own inhibitor, BIN1, in PC cells.

Furthermore, MYC is regulated and silenced by repressive signaling mediated by AR in the normal prostate but paradoxically is overexpressed and stimulated by AR in the early and late malignant phenotype in AR-driven PC [58,59]. MYC has been shown to confer resistance to androgen receptor signaling antagonists and promote androgen-independent growth of PC cells [60]. This apparent feature is significant for progression to end-stage disease where cells are selected/acquire resistance to AR signaling ablation and, as mentioned, CRPC often has elevated MYC and/or MYCN [21] expression.

## 3. MYC Cooperates with PIM Kinase Signaling in PC 

As discussed, there is overwhelming evidence that MYC is commonly dysregulated in PIN, carcinoma in situ (CIS) and metastatic, therapy-resistant CRPC by acting at least in part via increasing ribosome biogenesis in the cell. Acting in concert, MYC often collaborates with growth and pro-survival signaling pathways consistently upregulated in PC which act to increase the efficiency of mRNA translation, utilizing the newly synthesized ribosomes. Some of the most significant of these are the Proviral integration site for Moloney murine leukemia virus (*PIM*) kinases. PIMs are serine/threonine kinases which exist as three distinct paralogs [61] and are frequently overexpressed in prostate cancer and correlate with poor outcome [61,62]. They function through multiple signaling pathways by phosphorylating and inactivating multiple substrates implicated in PC progression. Accordingly, they have been shown to cooperate with MYC to promote survival signaling, and counteract MYC-driven apoptotic signaling [63,64], importantly phosphorylating and suppressing Bcl2-associated death promoter (BAD) [65]. In enhancing protein synthesis, PIM-2 is shown to inhibit 4E-BP1, an inhibitor of cap-dependent mRNA translation [64,66]. In the development of prostate cancer, overexpression of PIM-1 drives normal duct epithelium toward PIN lesions [67]. PIM kinases have also been reported to increase MYC protein stability by phosphorylating a regulatory residue serine 62 (Ser62) [68]. Convincingly, PIMs regulate MYC transcriptional activity through phosphorylation of S10 on histone H3 (p-HH3) [69]. Additionally, PIM-2 is known to be involved in phosphoinositol-3-kinase (PI3K) signaling by regulating mTORC1 activity via Tuberous Sclerosis Complex 2 (TSC2) suppression [70]. PIM-1 is also known to enhance transcriptional activity of the androgen receptor (AR) by phosphorylation of S213 and this remains prevalent in aggressive disease [71]. In considering all of these data, PIM kinases are unequivocally strong MYC partners in driving PC and represent a target which remains relevant in every stage of PC, including CRPC.

## 4. Mouse Models of MYC-Driven Prostate Cancer

In contrast to the zonal anatomy of the human prostate, the rodent prostate has distinct lobular divisions [6,8]. This consists of twin pairs of dorso-lateral, ventral and anterior lobes. Despite this difference, the cellular and molecular features in both the healthy function of the tissue and in pathological progression to cancer in genetically engineered models of disease (GEMM) are very similar [8]. This makes the mouse prostate an ideal model for human prostate cancer progression. There have been several mouse models generated over the past decade which have aimed to study the impact of MYC overexpression in the prostate (Table 1). Interestingly, the phenotypes of the mice are often dependent upon the status of additional disease drivers.

## 5. MYC-Driven Genetically Engineered Models of PC

There has been significant traction in prostate cancer research with the generation of recent MYC-driven autochthonous (tumor originating in situ) models of PC. These models are advantageous as they represent benign, early and late-stage disease according to gene dosage and/or latency. Consistent with MYC being a key driver of disease, MYC-driven prostate malignancy recapitulates human pathology closely [72].

Almost three decades ago, Thomson and colleagues [73] generated some of the first evidence of MYC involvement in PC, in situ*.* They successfully initiated lesion development by viral-mediated *MYC* gene transfer into fetal mouse urogenital sinus homogenates which were then grafted underneath the host mouse kidney capsule. This is a technique still used to successfully grow or reconstitute mouse and patient prostate tissue xenografts [74]. This experiment showed that MYC overexpression had the capacity to initiate hyperplasia specifically in prostate epithelium [73]. When combined with overexpression of RAS, a cooperating oncogene leading to increased ERK activity, lesions resembling carcinoma were frequently observed [73]. Using the same method, Wang and colleagues [75] more recently showed that MYC also synergizes with PIM-1 kinase to accelerate progression to cancer (Table 1). However, these cancers displayed mixed phenotype adenocarcinoma and NEPC, suggesting an unspecified cell of origin using this method of viral transduction mediated gene overexpression. Though powerful tools, these experiments highlight the importance of models of endogenously generated prostate cancer driven by germline transgene expression for reliable pathology.

The concept that MYC initiates neoplastic transformation in benign prostate epithelium is further evidenced by the probasin-directed models of prostate cancer. Probasin is an androgen-regulated gene essential for prostate development and was shown to be prostate epithelium-specific [79]. Rat probasin (Pbsn) promoters have been successfully used to direct expression of transgenes [80] including human MYC to the prostate. Ellwood-Yen and colleagues [72] demonstrated that MYC expression was sufficient to induce PC, characteristically resembling adenocarcinoma (Table 1). Pbsn-MYC (Lo-MYC) mice display nuclear atypia and HGPIN as their predominant pathology, but they progress to invasive adenocarcinoma at 10 months of age [50,72]. It is thought that MYC-induced genomic instability over time gives rise to the cancer phenotype and increasing nucleolar MYC expression shortens this latency [26]. The variant construct ARR2Pbsn-MYC (Hi-MYC) drives increased expression of MYC by the incorporation of additional androgen receptor response (ARR2) elements, which increase transgene expression within the prostate [72]. In this model, the high expression of MYC is also dependent upon AR to provide essential survival signaling, thus potentially overriding MYC-driven death signaling [72]. These mice present with invasive adenocarcinoma at as early as 3 months with 100% penetrance by 6 months, showing that MYC dosage accelerates progression of the disease [72]. Resulting tumors are highly vascularized, suggesting a role for MYC in angiogenesis although these mice have never been reported to show any metastatic foci. Furthermore, loss of ductal smooth muscle actin is a key feature of microinvasive adenocarcinoma and a differential feature in MYC overexpressed PC in GEMM [72]. More recently, MYC was shown to have a role in inducing smooth muscle cell de-differentiation and loss which may directly account for the microinvasive lesion phenotype and subsequent loss of duct integrity seen in Hi-MYC mice [81,82] as compared with its absence in low MYC models. Certainly, this is a significant aspect of the tumor for cells to achieve metastatic capability and progress toward end-stage disease rapidly.

For decades, therapeutic strategies have been developed for PC targeting a key feature of prostate cancer cell growth, hormone dependence. These therapies have proven effective in the initial treatment of prostate cancer as PC cells are unable to tolerate an acute loss of circulating testosterone or interrupted AR signaling and undergo apoptosis as a result [2]. However, it is now known that small populations of tumor cells will survive initial androgen ablation, are castrate tolerant and demonstrate tumor repopulating capacity [83]. The Hi-MYC and PTEN-null models also show that MYC-positive tumors can be resistant to castration-induced cell death [72,84], and may mimic the clinical outcome of therapy-resistant tissue accurately. Clinically, MYC overexpression is linked to biochemical recurrence (relapse detected through increased PSA expression) and progression to hormone refractory disease [30]. Certainly, MYC has a functional role in overcoming prostate epithelial cell androgen-dependence which is not fully delineated. This phenomenon is also seen in mouse models of MYC-driven PC, even though MYC is regulated by AR [85]. Kim and colleagues [77] provided strong evidence that MYC-driven prostate cancer is castrate resistant. In Z-MYC; Pbsn-Cre prostate lesions driven by MYC, MYC expression remains unaffected by castration and these lesions do not fully regress but instead become static [77]. This is also true of Hi-MYC [72] and HOXB13-MYC [76] mice. HOXB13 is a prostate-specific gene that is not androgen regulated. Therefore uncoupled from an AR regulated promotor, the transgene remains able to drive MYC post castration. Here, survival of MYC-dependent cells in CRPC may better model end-stage disease more accurately and drive development of future strategies for therapy in this setting.

## 6. Cooperative Oncogenic Signaling in GEMM

**Figure 1:**
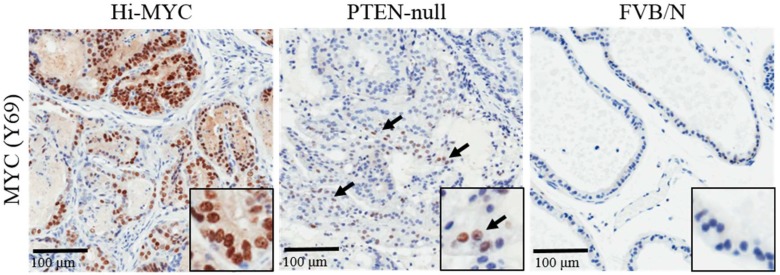
Expression levels of MYC in 5-month-old Hi-MYC (ARR2Pbsn-MYC), PTEN-null (PTEN^Fl/Fl^;Pbsn-Cre) and FVB/N (Wild type) prostate. MYC is overexpressed in Hi-MYC but also elevated in PTEN null prostate carcinoma.

Loss-of-function genetic aberrations affecting tumor suppressor genes are frequent drivers of PC. Accordingly, gene deletions occurring in PC have been successfully modelled in mice using Cre-Lox recombination. Probasin-Cre (PB-Cre) mice were generated to drive Cre recombinase expression under the control of the probasin promoter [86,87,88] and confirmed histopathological observations of carcinoma arising in P27^kip^/PTEN heterozygous loss of function GEMM with non-tissue-specific loss of PTEN function [89]. The PTEN-null mouse prostate is diffusely affected with carcinoma in situ (CIS) as the predominant lesion [86,87] (Table 1). Although tumorigenesis is driven by independent signaling via unregulated PI3K-AKT pathway activity, MYC is certainly upregulated in these tumors (Figure 1). Z-MYC;Pbsn-Cre conditional knockout mice develop focal HGPIN within prostatic ducts at 20 weeks. When crossed with PTEN^Fl/+^ mice, they show evidence of accelerated disease, adenocarcinoma, at 45 weeks where PTEN^Fl/+^ mice do not display pathology at all. This is further supported by the combinatorial model, Hi-MYC;PTEN^Fl/Fl^;Pbsn-Cre, which displays accelerated tumorigenesis [90]. Phosphoinositol-3-kinase (PI3K) pathway, which coordinates cell growth via AKT and mTORC1 activity, is widely active in prostate tumorigenesis [91] and is also implicated in MYC-driven prostate malignancy and therapeutic resistance [92]. Modelling AKT hyperactivity in mice with “constitutively active” AKT (MPAKT) forces progression to benign HGPIN without onset of carcinoma in 60-week-old mice [93]. Double transgenic Hi-MYC/MPAKT mice also demonstrate the ability of MYC to accelerate AKT-driven prostate tumor growth and this confers resistance to rapamycin treatment [90,94]. Taken together, this shows in vivo, that MYC cooperates with PTEN deficiency to accelerate carcinoma.

## 7. MYC in Non MYC-Initiated PC

Clearly, there is also a role for MYC upregulation driving disease in non MYC-initiated prostate malignancy. As mentioned, genomic aberrations surrounding the *MYC* locus often lead to MYC overexpression, however latent MYC dependency is a characteristic of tumors which have no observed genomic alterations. MYC protein overexpression and activity is shown in combinatorial B-Raf+;PTEN-null GEMM tumors which were not specifically designed to overexpress MYC [95]. Additionally, models of tumor suppressor p53 (TP53) and PTEN tumor suppressor co-deletion present with MYC-driven metastatic adenocarcinoma. Cho and colleagues show this in RapidCaP (lentiviral (LV)-Cre/Luci PTEN^Fl/Fl^;TP53^Fl/Fl^) mice [78]. Forty-five days after lentiviral intraprostatic injection of a vector containing Cre recombinase, transgenic mice develop focal CIS with reliable metastasis to lymph nodes, liver, lungs and kidney [78]. Though the original localized prostate tumor is AKT driven and maintains low MYC expression, curiously every metastasis displays strong MYC expression and reduced AKT activity [78] via PHLPP2-mediated suppression of AKT [96]. Therapeutic castration delayed but did not prevent growth of MYC-dependent metastasis [78]. This dependence on MYC was demonstrated by treatment with bromodomain extraterminal enhancer (BET) inhibitor, JQ1, post castration where CRPC tumors regressed and MYC expression was attenuated [78]. Although not designed as a MYC model, RapidCaP features high penetrance of the MYC metastatic phenotype which demonstrates a short latency to end stage disease.

Another androgen-regulated, prostate-specific protein, NKX3.1, is frequently used to model PC. NKX3.1 is a homeobox (HOX) gene specifically expressed in the prostate and seminal vesicle which is thought to provide significant tumor suppressor function in the adult prostate in PTEN-null prostate tumors [97,98,99]. The generation of NKX3.1 directed expression of Cre recombinase showed similar pathology as Pbsn-Cre;PTEN^Fl/Fl^ mice whereby carcinoma in situ results from deletion of PTEN alone. A number of models have shown that additional deletion of TP53 drives metastatic disease [78,100,101] and strikingly, that MYC overexpression appears to be a key feature of tumors with PTEN deletion [95,101,102]. Iwata and colleagues [50] attempted to model NKX3.1-directed overexpression of MYC in mice. These “super” Lo-MYC mice displayed HGPIN. Paradoxically, the transgene expression enforced its own downregulation via MYC repression of NKX3.1 stability [50]. As a result MYC expression induced focal LGPIN lesions as its only pathology. Certainly, one major function of MYC is to specifically downregulate NKX3.1 at the protein level [49] but how this is exactly achieved remains unknown. Nevertheless, loss of NKX3.1 is integral to MYC-driven malignancy of the prostate and may deregulate NKX3.1 control of PTEN-null cells to accelerate PC.

## 8. Overcoming AR Regulation in PC Modeling

A major limitation to modelling prostate cancer therapeutic efficacy in vivo is that most avenues to direct transgene expression to the prostate epithelium involve gene promoters which are under the control of the androgen receptor and as a result, experimentally mimicking androgen deprivation therapy (ADT) without affecting transgene expression is not feasible. Hubbard and colleagues recently generated and described a model directed by HOXB13 which is androgen-independent but prostate specific [76]. By four weeks, HOXB13-MYC;PTEN^Fl/Fl^ (BMPC) mice display AR positive PIN lesions with high MYC expression and combined PTEN deletion [76]. Invasive adenocarcinoma is reached by 4 months, with the loss of AR expression, NKX3.1 and near-complete penetrance of lymph node metastasis with liver and lung among the most prevalent subsequent sites of metastasis [76]. Castration of BMPC mice limited spread of the tumor but ultimately had limited therapeutic effect [76]. As mentioned, this resistance to therapeutic castration appears to be a common feature of MYC-driven GEMM and interestingly, in BMPC mice and others (RapidCaP, Hi-MYC;PTEN-null, Z-MYC;PTEN-null), additional PTEN [90] and/or TP53 [78] loss of heterozygosity are requirements for MYC-driven metastasis. Mechanistically, there could be a link here between MYC-induced genomic instability and cooperativity with the recently-described role for nuclear PTEN deficiency inducing genomic instability [103]. These are significant observations considering the frequency of these co-occurring gene aberrations in therapy-resistant metastatic CRPC [104]. This relationship requires further thought but through the careful and differential analysis of MYC-driven GEMM, MYC unequivocally remains prevalent in CRPC.

## 9. Using MYC-Driven PC Models to Test Novel Therapeutics

Primary therapy for the treatment of PC includes surgical excision of the prostate (radical prostatectomy) and radiotherapy [3]. Recurrent PCs which can arise from residual tissue due to intrinsic or acquired treatment-induced resistance are treated with androgen deprivation therapy through surgical or chemical castration which is effective in bringing about sustained remission [3]. A second relapse is invariably castrate-resistant but responds to further AR blockade initially. Efficacy has been seen with new line AR signaling antagonists such as abiraterone acetate that target the biosynthesis of testosterone [105] or enzalutamide, which inhibits translocation of AR to the nucleus and also reduces AR transcriptional activity [106]. Even still, the continued use of hormone-based therapy only adds a few months of life and tumor growth despite AR blockade is frequently observed. In this context of androgen-independent growth, taxane-derived chemotherapies such as docetaxel and cabazitaxel have been shown to improve therapeutic outcome [107]. The high toxicity and low selectivity of these compounds is a key issue in the treatment of metastatic CRPC and as discussed, PC has a distinct profile of MYC expression which dominates the therapy-resistant phenotype. Therefore, it is essential to explore the development of novel targeted agents which address the key pathways commonly activated in CRPC such as MYC family overexpression.

## 10. Targeting Transcription of MYC

The development targeted agents against MYC have been hindered by challenges associated with its structure and function. MYC protein lacks enzymatic activity and functions primarily via protein-DNA and protein-protein interactions which make its structure difficult to design inhibitory small molecules [108]. As briefly described above, there is a major role for MYC in chromatin remodeling and this effect is partly mediated through bromodomain extraterminal enhancer (BET) proteins [109]. Bromodomains are regulatory regions which function by recognizing acetyl-lysine modifications on histones [110]. There are four paralogs in the BET family of proteins, BRD2, BRD3, BRD4 and BRDT [110]. The aberrant MYC-driven oncogenic signaling induces hyperacetylation and BET family-mediated signal transduction and carcinogenesis [109]. In recent years, BET bromodomain inhibitors have demonstrated preclinical efficacy in models of MYC-driven CRPC by targeting upstream of MYC [111,112], disrupting BRD4/AR interactions and subsequent AR-directed transcription of MYC. JQ1 and I-BET762, novel BRD4 bromodomain inhibitors, were shown to reduce the expression of MYC in pre-clinical models of PC. This correlated with the marked inhibition of cell growth and induction of cell death in PC cells [111]. Inhibiting BET bromodomains also inhibited MYC-dependent RNA pol II function which enhanced this antiproliferative effect. BET inhibition represents a promising mode of MYC inhibition for prostate cancer therapy at the level of suppression of MYC mRNA transcription.

## 11. Targeting MYC Function

Significant to activity of MYC is its binding with MYC-associated protein X (MAX), forming a heterodimer which preferentially binds E-box motifs. As discussed, in the context of the cancer cell this transcription factor complex binds target gene promotors in order to stimulate or repress transcriptional activity, thereby deregulating cell growth. Therefore, studies have evaluated the efficacy of disrupting this relationship with MAX with the dominant-negative MYC-binding compound, Omomyc [113,114,115], and more recently with KJ-Pyr-9 [116] which interferes with the MYC-MAX protein-protein interaction. Further study with these compounds will determine whether they have robust in vivo effects in models of prostate cancer.

As discussed, MYC displays diverse and multifaceted pro-tumorigenic effects in the initiation and development of PC whereby deregulation of ribosome biogenesis and function via cooperation with PIM overexpression is dominant and prevails through to advanced disease. Recognizing this quality of MYC, we previously demonstrated that Pol I inhibition was effective in MYC-driven hematological malignancy and synergized with inhibitors of mRNA translation to extend the therapeutic effect. The Pol I inhibitor, CX-5461, initiated TP53-dependent cell death in Eµ-MYC lymphoma [117]. Combination with the mTORC1 inhibitor, everolimus, potentiated the effect via Bcl-2-Modifying Factor (BMF) to initiate cell death in Eµ-MYC cells [118]. We also show that CX-5461 limited PC growth in Hi-MYC mice, however in this context there is an absence of rapid cell death [119]. We suspected that CX-5461 in a similar combination which targets ribosome function may be effective in enhancing this effect in PC.

Recently, the dependence of MYC on PIM for function has been noticed increasingly in models of PC. Kirchner and colleagues utilized MYC/PIM transduced tissue recombination model showing that inhibition of PIM with pan-PIM kinases inhibitor, AZD-1208, was effective in limiting MYC-driven lesion progression [120]. Mechanistically, AZD1208 administration suppresses 4E-BP1 phosphorylation and prevents cap-dependent translation of mRNAs but also initiates downstream suppression of mTORC1 via TSC2 phosphorylation inhibition [121], thus inhibiting PI3K pathway activity as well. This inhibition efficacy was also demonstrated in MYC-CaP cell allografts, a cell line generated from Hi-MYC mice. Predictably, AZD1208 induces MYC de-stabilization by inhibiting S62 phosphorylation and stability of MYC [120]. Additionally, AZD-1208 relieves PIM-mediated BAD activity suppression by phosphorylation of S112 [120]. PIM is also synthetic lethal in models of MYC-dependent, hormone naive breast cancer [122] and small-molecule PIM inhibitors are effective in limiting MYC-driven breast cancer cell growth in vivo [123]. Building on these observations, we have demonstrated that PIM inhibition with pan-PIM inhibitor, CX-6258, cooperates with targeted Pol I therapy and is effective in treating MYC-driven prostate malignancy [119]. Hi-MYC mice that were treated with CX-5461, an RNA Pol I inhibitor, show marked reduction in proliferative index and incidence of tumor foci [119]. This is mediated by nucleolar stress leading to the accumulation of TP53 and subsequent cell cycle arrest. Models of PC without intact TP53 pathway were also shown to be sensitive to this therapy. These data suggest that MYC-dependent PC relies heavily on ribosome biogenesis. Combined Pol I and PIM inhibition further suppressed Hi-MYC tumorigenesis and demonstrated efficacy in PTEN-null and high-MYC patient-derived xenograft (PDX) models of prostate cancer [119]. Other MYC-driven solid tumors have also recently been shown to have exquisite sensitivity to Pol I inhibition. MYC, MYCN and MYCL-driven autochthonous lung tumors in GEMM have stunted growth after CX-5461 administration [124]. This work invites further pre-clinical validation by the use of sophisticated models of patient-derived PC such as prostate organoids [125] and PDX of CRPC [74] which may provide additional insight to observations made in GEMM. Nevertheless, these recent data demonstrate the potential for targeted therapy downstream of MYC in advanced CRPC, targeting both ribosome synthesis and function and thus providing an exciting new avenue for therapy which does not follow traditional routes.

## 12. Final Remarks

Prostate cancer research has been accelerated by the use of GEMM, which generate tumors in situ from untransformed mouse prostate tissue. MYC is a clear oncogenic driver of advanced PC but targeting MYC directly has proven difficult due to its structure and function as a transcription factor. Strategies which target synthesis of MYC via BET inhibition or downstream signaling effectors such as Pol I and PIM which target both ribosome synthesis and function, remain attractive modes for MYC-targeted therapy in PC. BET, Pol I and PIM inhibitors all remain in clinical trials.

## Figures and Tables

**Table 1:** Mouse models of MYC-driven prostate cancer.

Expression of MYC	Model	Transgene	Pathology and Timecourse	Driving Mutations	Reference
Local: + + + + Mets: + + + +	BMPC	HOXB13-MYC; HOXB13Cre; PTEN^Fl/Fl^	Diffuse HGPIN < 2 months IAC = 3–5 months Mets to lymph, liver, lung and bone > 5 months	MYC overexpression; PTEN deletion	[76]
+ + + +	Hi-MYC	ARR2Pbsn-MYC	Diffuse HGPIN < 3 months Focal IAC > 3 months	MYC overexpression	[72]
+ + +	Lo-MYC	Pbsn-MYC	Diffuse HGPIN < 10 months Focal IAC > 10 months	MYC overexpression	
+ + + +	MYC/PIM-1 Recombinant	LV-MYC/PIM-1	Focal NEPC > 1.5 months/Focal IAC > 1.5 months	MYC overexpression; PIM-1 overexpression	[75]
+ + +	MYC Recombinant	LV-MYC	Focal IAC > 3 months	MYC overexpression	
+ + +	Z-MYC	Pbsn-Cre; MYC	Focal LGPIN > 12 months	MYC overexpression	[77]
+ + +	Z-MYC /PTEN-het	Pbsn-Cre; MYC; PTEN^Fl/+^	Focal HGPIN < 11 months. Focal IAC > 11 months	MYC overexpression; PTEN single allele deletion	
+ + +	Z-MYC /PTEN-null	Pbsn-Cre; MYC; PTEN^Fl/Fl^	Focal IAC > 2 months	MYC overexpression; PTEN deletion	
Local: + Mets: + + +	Rapid-CaP	LV-Cre/Luciferase PTEN^Fl/Fl^; TP53^Fl/Fl^	Focal CIS > 1.5 months Mets to lymph, liver, lung and kidney > 1.5 months	PTEN deletion; TP53 deletion	[78]
+ +	“Super” Lo-MYC	MYC/KAN-NKX-3.1	LGPIN > 2 months	MYC overexpression	[50]

Mets: Metastasis; NEPC: neuroendocrine prostate cancer; PIN: prostatic intraepithelial neoplasia; HGPIN: high-grade PIN; LGPIN: low-grade PIN; CIS: carcinoma in situ; IAC: invasive adenocarcinoma; Pbsn: probasin; TP53: tumor suppressor p53; LV: lentiviral.

## Abbreviations

ADT	Androgen deprivation therapy
AR	Androgen receptor
BET	Bromodoman extraterminal enhancer
CIS	Carcinoma in situ
CRPC	Castrate resistant prostate cancer
GEMM	Genetically engineered mouse model
HGPIN	High grade PIN
IAC	Invasive adenocarcinoma
LV	Lentiviral
LGPIN	Low grade PIN
NEPC	Neuroendocrine Prostate Cancer
Pbsn	Probasin
PC	Prostate cancer
PDX	Patient-derived xenograft
PI3K	Phosphoinositol-3 kinase
PIN	Prostatic intraepithelial neoplasia
Pol	RNA Polymerase
TP53	Tumor suppressor p53
TSG	Tumor suppressor gene
